# Supplementary material for: Massive and rapid COVID-19 testing is feasible by extraction-free SARS-CoV-2 RT-PCR
Source: Nat Commun. 2020 Sep 23;11:4812. doi: 10.1038/s41467-020-18611-5 (PMC7511968; doi:10.1038/s41467-020-18611-5)
Supplement: Supplementary file 1 — Supplementary Information [file 41467_2020_18611_MOESM1_ESM.pdf]

SUPPLEMENTARY INFORMATION

Massive and rapid COVID-19 testing is feasible by extraction-free SARS-CoV-2 RT-PCR

Ioanna Smyrlaki[1,*], Martin Ekman[2,*], Antonio Lentini[1], Nuno Rufino de Sousa[3], Natali Papanicolaou[1], Martin Vondracek[2], Johan Aarum[2], Hamzah Safari[2], Shaman Muradrasoli[4], Antonio Gigliotti Rothfuchs[3], Jan Albert[2,3], Björn Högberg[1] and Björn Reinius[1,#]

[1] Department of Medical Biochemistry and Biophysics, Karolinska Institutet, Stockholm, Sweden.

[2] Department of Clinical Microbiology, Karolinska University Hospital, Stockholm, Sweden.

[3] Department of Microbiology, Tumor and Cell Biology, Karolinska Institutet, Stockholm, Sweden.

[4] Public Health Agency of Sweden, Solna, Sweden.

* Equal contribution

# Correspondence to bjorn.reinius@ki.se

Supplementary Figures 1-2
Supplementary Tables 1-3

Supplementary figure 1

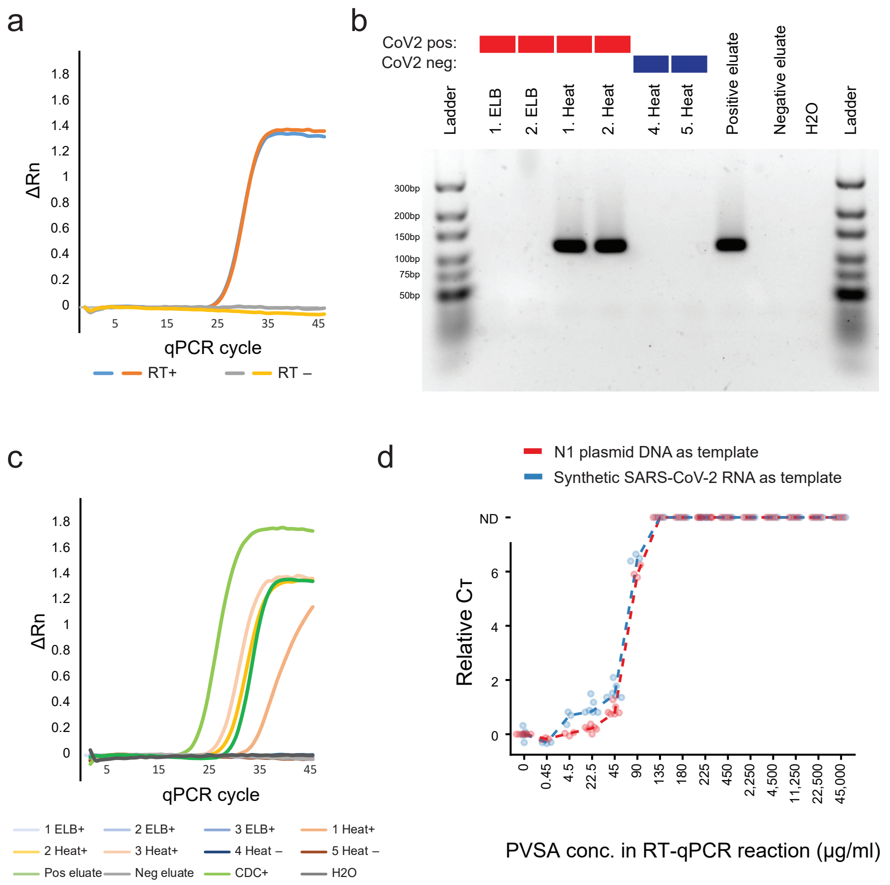

Supplementary figure 1. (**a**) Amplification plots showing normalized reporter value (ΔRn, linear scale) as a function of qPCR cycle for two-step RT and qPCR performed on synthetic full-genome SARS-CoV-2 RNA (SKU102024-MN908947.3, Twist Biosciences) including (RT+) or excluding (RT-) the reverse transcriptase. (**b**) Agarose gel electrophoresis of RT-PCR products for reactions described in Fig.2e, but in this case using primers and probes for gene E. (**c**) Amplification plots showing normalized reporter value (ΔRn, linear scale) as a function of qPCR cycle for the samples described in Fig. 2e-f, but using two-step RT and qPCR instead of the single-step TaqPath RT-qPCR (Methods). (**d**) Relative cycle threshold (C_T) values of SARS-CoV-2 RT-PCR using the N1 primer-probe set and a dilution series of the chemical RNase inhibitor polyvinylsulfonic acid (PVSA). To test the inhibition of PVSA in RT and PCR reactions separately, we used either synthetic full-genome SARS-CoV-2 RNA (blue dots and line line) or plasmid DNA (CDC positive control) (blue dots and line) as template in the RT-PCR reaction. C_T values are shown relative to reactions without PVSA (conc.= 0). The lines indicate the median of triplicate measurements.

Supplementary figure 2

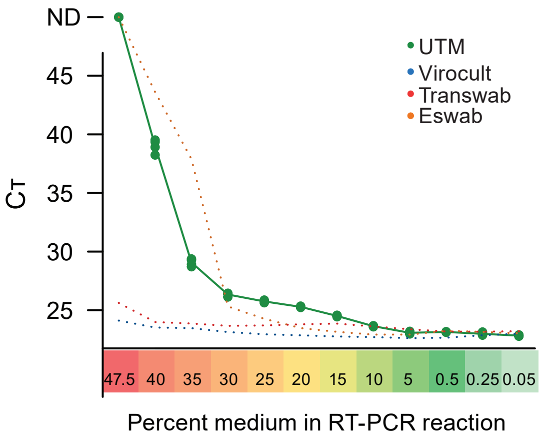

Supplementary figure 2. C_T values from RT-PCR performed on dilution series of Universal Transport Medium (UTM, Copan) using 50,000 spiked copies of synthetic full-genome SARS-CoV-2 RNA and the N1 primer-probe set. Lines represent the mean and the values of individually replicates (n=4) are shown as dots. Mean values for Virocult, Transwab and Eswab are shown as dashed lines, included for comparison.

Supplementary table 1

Supplementary table 1. C_T values for 21 clinical nasopharyngeal swab samples quantified using cobas 6800 and TaqPath SARS-CoV-2 RT-PCR on eluted RNA. We observed dropout of detection in four positive samples in RT-PCR on eluted RNA. ND: Not detected (C_T >40) ViCu: Virocult. TSw: Transwab.

**Sample medium**	**cobas 6800**		**RT-PCR (eluate)**	
	**ORF1**	**E**	**RdRP**	**E**
ViCu	32.83	33.89	32.50	34.28
ViCu	22.95	23.10	18.57	18.12
ViCu	32.08	33.15	32.47	33.84
ViCu	30.23	30.95	29.36	29.94
TSw	30.67	31.54	30.63	30.47
ViCu	34.89	37.65	35.77	ND
ViCu	20.22	20.29	17.45	17.35
TSw	24.35	24.50	24.14	23.46
TSw	25.98	26.27	26.05	25.17
TSw	33.92	35.88	ND	33.94
TSw	23.95	24.09	23.33	22.59
TSw	27.33	27.61	26.62	26.32
NA	32.24	33.25	34.28	32.69
NA	33.02	33.95	ND	ND
NA	33.08	34.31	33.54	33.55
ViCu	ND	ND	ND	ND
ViCu	ND	ND	ND	ND
ViCu	ND	ND	ND	ND
ViCu	ND	ND	ND	ND
ViCu	ND	ND	ND	ND
ViCu	ND	ND	ND	ND

Supplementary table 2

Supplementary table 2. Limit-of-detection data. C_T values for a COVID-19 positive clinical nasopharyngeal swab sample diluted 1:100, 1:1000, 1:10,000 or 1:100,000 in PBS and quantified using cobas 6800 and TaqPath SARS-CoV-2 RT-PCR on eluted RNA.

**Sample dilution**	**Replicate**	**cobas 6800**	**RT-PCR (eluate)**	**cobas 6800**	**RT-PCR (eluate)**
		**E**	**E**	**ORF1**	**RdRP**
1:100	1	35.16	34.98	35.14	33.18
	2	34.97	ND	34.95	33.89
	3	35.26	32.86	34.60	32.92
1:1000	1	37.35	36.53	36.32	33.90
	2	36.25	35.36	36.06	34.55
	3	36.68	ND	35.88	35.36
1:10,000	1	37.91	35.31	37.05	35.55
	2	38.71	ND	ND	ND
	3	39.09	ND	ND	ND
1:100,000	1	ND	ND	ND	ND
	2	ND	ND	ND	ND
	3	ND	ND	ND	ND

Supplementary table 3

Supplementary table 3. Generic buffers evaluated for SARS-CoV-2 hid-RT-PCR.

Buffer	Composition	Measured pH	Company catalog number (if available)
PVSA in H_2O pH6.5	Polyvinylsulfonic acid in H_2O (50µg/ml)	6.5	Sigma 278424, Ambion AM9932
TE + 4% sucrose pH7.0	TE (10 mM Tris; 0.1mM EDTA) + 4% sucrose	7.0	Invitrogen AM9858
Tris buffer 10mM pH7.0	10mM Tris buffer	7.0	
PVSA in Tris buffer pH7.0	PVSA in 10mM Tris buffer (50µg/ml PVSA)	7.0	
Tris-HCl 5mM pH7.4	Tris-HCl 5mM	7.4	
TE pH7.0	TE (10 mM Tris + 0.1mM EDTA)	7.0	Invitrogen AM9858
PBS 10mM pH7.5	10mM PBS	7.5	Invitrogen AM9624
Sodium citrate 1mM pH7.6	1mM Sodium citrate	7.6	
H_2O	H_2O	6.4	Ambion AM9932
PVSA in 1mM Sodium citrate pH7.5	PVSA in 1mM Sodium citrate (50µg/ml PVSA)	7.5	
TE + 4% sucrose pH8.0	TE (10 mM Tris; 0.1mM EDTA) + 4% sucrose)	8.0	Invitrogen AM9858
TE pH8.1	TE (10 mM Tris 0.1mM EDTA)	8.1	Invitrogen AM9858
HBSS pH8.4	HBSS (Hanks' Balanced Salt solution)	8.4	Gibco 14025-092
PBS 100mM pH7.7	100mM PBS	7.7	Invitrogen AM9624
Saline 0.9% pH5.8	0.9% NaCl in 1L water	5.8	
DMEM pH7.9	DMEM (Dulbecco's Modified Eagle Medium)	7.9	Sigma D5796
Citrate buffer pH6.2	Citrate buffer (0.04M Sodium citrate dihydrate; 0.06M Citric acid)	6.2	
