# Supplementary material for: Massive and rapid COVID-19 testing is feasible by extraction-free SARS-CoV-2 RT-PCR
Source: Nat Commun. 2020 Sep 23;11:4812. doi: 10.1038/s41467-020-18611-5 (PMC7511968; doi:10.1038/s41467-020-18611-5)
Supplement: Supplementary file 3 — Reporting Summary [file 41467_2020_18611_MOESM3_ESM.pdf]

nature research

Corresponding author(s): Björn Reinius

Last updated by author(s): Aug 5, 2020

Reporting Summary

Nature Research wishes to improve the reproducibility of the work that we publish. This form provides structure for consistency and transparency in reporting. For further information on Nature Research policies, see our Editorial Policies and the Editorial Policy Checklist.

Statistics

For all statistical analyses, confirm that the following items are present in the figure legend, table legend, main text, or Methods section.

n/a	Confirmed	
☐	☒	The exact sample size (*n*) for each experimental group/condition, given as a discrete number and unit of measurement
☐	☒	A statement on whether measurements were taken from distinct samples or whether the same sample was measured repeatedly
☐	☒	The statistical test(s) used AND whether they are one- or two-sided<br>*Only common tests should be described solely by name; describe more complex techniques in the Methods section.*
☐	☒	A description of all covariates tested
☐	☒	A description of any assumptions or corrections, such as tests of normality and adjustment for multiple comparisons
☐	☒	A full description of the statistical parameters including central tendency (e.g. means) or other basic estimates (e.g. regression coefficient) AND variation (e.g. standard deviation) or associated estimates of uncertainty (e.g. confidence intervals)
☐	☒	For null hypothesis testing, the test statistic (e.g. *F*, *t*, *r*) with confidence intervals, effect sizes, degrees of freedom and *P* value noted<br>*Give P values as exact values whenever suitable.*
☒	☐	For Bayesian analysis, information on the choice of priors and Markov chain Monte Carlo settings
☒	☐	For hierarchical and complex designs, identification of the appropriate level for tests and full reporting of outcomes
☒	☐	Estimates of effect sizes (e.g. Cohen's *d*, Pearson's *r*), indicating how they were calculated

Our web collection on statistics for biologists contains articles on many of the points above.

Software and code

Policy information about availability of computer code

Data collection	*Provide a description of all commercial, open source and custom code used to collect the data in this study, specifying the version used OR state that no software was used.*
Data analysis	cobas 6800 instrument software version used was 01.03.08.1011. The Step-One-Plus real time PCR machine was operated using the StepOne Software v2.3. R computer code is available at https://github.com/reiniuslab/COVID19.

For manuscripts utilizing custom algorithms or software that are central to the research but not yet described in published literature, software must be made available to editors and reviewers. We strongly encourage code deposition in a community repository (e.g. GitHub). See the Nature Research guidelines for submitting code & software for further information.

Data

Policy information about availability of data

All manuscripts must include a data availability statement. This statement should provide the following information, where applicable:

- Accession codes, unique identifiers, or web links for publicly available datasets
- A list of figures that have associated raw data
- A description of any restrictions on data availability

Source data are provided with the paper, as well as on https://github.com/reiniuslab/COVID19.

Field-specific reporting

Please select the one below that is the best fit for your research. If you are not sure, read the appropriate sections before making your selection.

☒ Life sciences ☐ Behavioural & social sciences ☐ Ecological, evolutionary & environmental sciences

For a reference copy of the document with all sections, see nature.com/documents/nr-reporting-summary-flat.pdf

Life sciences study design

All studies must disclose on these points even when the disclosure is negative.

Sample size	Sample size for the benchmarking (n= 597 patient samples) is sufficient as it covers the spectra of Ct values detected in patient samples on the cobas 6800 (See Fig. 5a-c) and the distribution is not different from a very large number of samples (n= 9437) historically collected on the same machine.
Data exclusions	No data was excluded
Replication	Replicate measurement was performed for experiments in the following figures and Ct values were highly similar: Fig. 2a (n=2), Fig. 2b (n=2), Fig. 4 (n= 9), Fig. 6a-b (n= 3), Fig. 7a-b (n= 3), Supplementary Fig. 1a (n=2), Supplementary Fig. 2 (n=4).
Randomization	Samples were tested blindly and positive/negative samples were randomly distributed over plates during testing.
Blinding	Samples were tested blindly

Reporting for specific materials, systems and methods

We require information from authors about some types of materials, experimental systems and methods used in many studies. Here, indicate whether each material, system or method listed is relevant to your study. If you are not sure if a list item applies to your research, read the appropriate section before selecting a response.

Materials & experimental systems

n/a	Involved in the study	
☒	☐	Antibodies
☐	☒	Eukaryotic cell lines
☒	☐	Palaeontology and archaeology
☒	☐	Animals and other organisms
☐	☒	Human research participants
☒	☐	Clinical data
☒	☐	Dual use research of concern

Methods

n/a	Involved in the study	
☒	☐	ChIP-seq
☒	☐	Flow cytometry
☒	☐	MRI-based neuroimaging

Eukaryotic cell lines

Policy information about cell lines

Cell line source(s)	Vero E6 cells were used for in vitro expansion of SARS-CoV-2. The line was a gift from The Public Health Agency of Sweden. The original Vero cell line was derived from kidney epithelial cells extracted from an African green monkey (Chlorocebus sp.), 1962 by Yasumura and Kawakita at the Chiba University in Chiba, Japan.
Authentication	The Vero E6 line was not independently authenticated. Vero E6 cells were used for in vitro production of SARS-CoV-2 and not for study of the cell line per se.
Mycoplasma contamination	Cell were tested negative to mycoplasma.
Commonly misidentified lines (See ICLAC register)	*Name any commonly misidentified cell lines used in the study and provide a rationale for their use.*

April 2020

Human research participants

Policy information about studies involving human research participants

Population characteristics	Persons in the Stockholm area, Sweden, tested either clinically for COVID-19 or participating in a COVID-19 screen.
Recruitment	In this work we used anonymized or pseudo-anonymized surplus material from samples that had been collected for clinical diagnostics of SARS-CoV-2 (Karolinska University Hospital, Stockholm, Sweden), in accordance with the Swedish Act concerning the Ethical Review of Research Involving Humans which allows development and improvement of diagnostic assays using patient samples which were collected to perform the testing in question. The samples used in direct lysis experiment (Fig. 7e) were deidentified self-collected volunteer samples from a COVID-19 clinical screen performed in the Stockholm area, organized by the Public Health Agency of Sweden.
Ethics oversight	Ethical approval was obtained by the appropriate Swedish Authority (Etikprövningsnämnden).

Note that full information on the approval of the study protocol must also be provided in the manuscript.
